# Early transcriptomic host response signatures in the serum of dengue patients provides insights into clinical pathogenesis and disease severity

**DOI:** 10.1038/s41598-023-41205-2

**Published:** 2023-08-29

**Authors:** Aanchal Yadav, Uzma Shamim, Varsha Ravi, Priti Devi, Pallawi Kumari, Ranjeet Maurya, Poonam Das, Madhuri Somani, Sandeep Budhiraja, Bansidhar Tarai, Rajesh Pandey

**Affiliations:** 1 Division of Immunology and Infectious Disease Biology, INtegrative GENomics of HOst-PathogEn (INGEN-HOPE) Laboratory, CSIR-Institute of Genomics and Integrative Biology (CSIR-IGIB), North Campus, Near Jubilee Hall, Mall Road, Delhi, 110007 India; 2 Academy of Scientific and Innovative Research (AcSIR), Ghaziabad, 201002 India; 3 Max Super Speciality Hospital (A Unit of Devki Devi Foundation), Max Healthcare, Delhi, 110017 India

**Keywords:** Viral infection, Viral infection

## Abstract

Dengue virus (DENV), known to cause viral infection, belongs to the family Flaviviridae, having four serotypes (DENV1-4) that spreads by the bite of the *Aedes aegypti* mosquito. India has been suffering from dengue outbreaks annually with widespread epidemics by prevalence of all the four DENV serotypes. The diverse spectrum of clinical manifestations in dengue infection, mild to severe forms, makes the need of timely diagnosis and prompt treatment an essence. The identification of a dengue host response signature in serum can increase the understanding of dengue pathogenesis since most dengue NS1 Ag tests have been developed and evaluated in serum samples. Here, to understand the same, we undertook a dual RNA-sequencing (RNA-Seq) based approach from the serum samples of dengue-infected patients. The results thus yield the early transcriptional signatures that discriminated the high viral reads patients from patients who had low dengue viral reads. We identified a significant upregulation of two sets of genes, key antiviral (*IFIT3*, *RSAD2*, *SAT1*) and vascular dysfunction (*TNFS10*, *CXCL8*) related genes in the high viral reads group. Deeper delving of this gene profile revealed a unique two-way response, where the antiviral genes can mediate the disease course to mild, contrarily the increased expression of the other gene set might act as pointers of severe disease course. Further, we explored the hematologic parameters from the complete blood count (CBC), which suggests that lymphocytes (low) and neutrophils (high) might serve as an early predictor of prognosis in dengue infection. Collectively, our findings give insights into the foundation for further investigation of the early host response using the RNA isolated from dengue patients’ serum samples and opens the door for careful monitoring of the early clinical and transcriptome profiles for management of the dengue patients.

## Introduction

Dengue infection is a common arthropod-borne virus disease, causing endemics in subtropic and tropic regions globally, especially India. It causes an acute febrile illness known as dengue fever (DF), which can progress to life-threatening severe forms of infection such as dengue haemorrhagic fever (DHF) and dengue shock syndrome (DSS) (World Health Organization. (1997). Dengue haemorrhagic fever: diagnosis, treatment, prevention and control. World Health Organization^1^). Due to low observed sensitivity of this classification system to detect severe cases of Dengue, WHO in 2009, developed a new classification of DF^2,3^. The revised classification segregated dengue into two major categories: non-severe and severe dengue (SDF); the non-severe dengue was further divided into dengue with warning signs (D + W) and dengue without warning signs (D-W). All these categories were created based on level of clinical severity as identified through a series of clinical symptoms listed for each group^4^. According to WHO, an outbreak of DENV infections is predicted to cause 15 million infections, resulting in 2 million cases of severe illness and 21,000 deaths^5^.

The dengue virus primarily consists of four genetically and antigenically diverse serotypes (DENV1-4) that circulate in endemic areas and cause infection. The Dengue serotypes, I-IV shows 65% genome sequence similarity while containing hypervariable regions distinguishing different serotypes. These hypervariable regions are present in E, NS2A, NS4 and NS5 coding regions of the Dengue genome, determining the genetic differences between the serotypes^6^. Infection by any of the serotypes manifest a wide range of clinical outcomes, from asymptomatic DENV infection, to mild DF and severe forms with DHF and DSS which can be fatal. The prevalence of life threatening severe dengue is comparatively low, characterized by plasma leakage, haemoconcentration, thrombocytopenia and abnormalities in homeostasis^7^. This heterogeneity of symptoms necessitates an understanding of the underlying cause for the symptomatic range. What are the triggering responses within the host that deviates clinical manifestation towards severity? Also, there are no clear-cut hallmarks to assist diagnosis, along with therapeutic challenges to fight the dengue virus. Studies have examined viral as well as host factors in defining dengue infection and its influence on clinical phenotypes that may be used as biomarkers of severity. Number of studies show that severity of disease can be influenced by different virus serotypes^8,9^. Patients with DHF were discovered to have higher viral loads compared to DF. In terms of serotype, DENV-2 is associated with severe forms of DHF^10^. Interestingly, dengue disease severity can be evaluated through clinical parameters, which has been considered as the primary sensitive method. It includes laboratory tests such as complete blood count (CBC), serological test or blood culture which can be used along with viral antigen positivity to confirm the diagnosis. In retrospective studies, the authors have identified CBC parameters along with other clinical parameters to differentiate various degrees of illness^11,12^. An abnormal platelet count and function has been labelled as a hallmark of dengue infection, yet there are other important parameters such as high atypical lymphocyte percent that are associated with differential disease severity^13^.

Considering the variation in viral genotype and clinical parameters among the dengue patients, numerous studies have suggested host immune response as important factors associated with the disease severity. Robinson et. al. identified 20 genes that predict progression to severe dengue using expression datasets from patients with acute dengue infection^14^. Consequently, the expression of several genes were identified to differ between DF and DHF patients, leading to progression of severe disease. Following that, genes such as *CFD, PSMB9, FCGR3B, MAGED1*, and *PRDX4* were differentially expressed in Dengue patients with mild or severe symptoms^15^. Evidence demonstrates that serum immune profiles of dengue patients shed light on the role of several serum cytokines in progression of disease and are found consistent with blood profiles^16^. Taken together, although several studies have reported host immune response that distinguishes mild phenotype of DF from severity in DHF, yet there is still a dearth of knowledge as to whether the direction of clinical progression could be assessed during early stages of infection? Moreover, serum NS1 antigen testing importantly helps to detect viral load of Dengue infection, which in turn affects disease severity. Henceforth, it seems imperative to look at the early host response in serum samples of Dengue patients collating together host and pathogen perspective from the same source i.e. serum samples. An initial study has analysed the host transcriptomic profile from serum samples of dengue positive patients^17^.

In our study, we characterized the Dengue disease pathogenesis from serum sample of patients, collected early during infection when a person is positive for NS1 antigen test. The clinical parameters are also simultaneously captured. Dual RNA-Seq enabled us to understand the host–pathogen interaction as well as clinical parameters using serum samples from dengue patients with high or low viral reads to elucidate underlying factors to disease illness. Our findings indicate that clinically, lymphocytes and neutrophils are important blood markers that can be predictively associated with differential disease severities. Moreover, host immune responses are differentially regulated between dengue patients with high and low viral reads. The accumulated evidence from our genomics-based study shed light on initial pathophysiology of dengue infection and integrative role of several factors in governing severity of Dengue infection.

## Results

### Patient demographics and clinical clustering of patients for functional analysis

Our study includes 24 serum samples which were collected from patients reporting to the OPD of MAX Healthcare hospital, Delhi, India with febrile illness. All these patients tested positive for NS1 ELISA for Dengue viral infection. The demographic characteristics of all the patients are summarized in Table 1. The age of the patients in the study cohort ranged from 3 to 68 years with a median age of 21.5 years. There was a preponderance of male patients, 66.7% (n = 16) compared to females 33.3% (n = 8).

**Table 1:** Summary of demographic characteristics of the 24 Dengue NS1 positive patients.

Variables	N = 24
Age (Y)	
Min	3
Max	68
Median	21.5
Gender	
Female	8 (33.3%)
Male	16 (66.7%)
NS1 Ag, median	3.45

**Figure 1:**
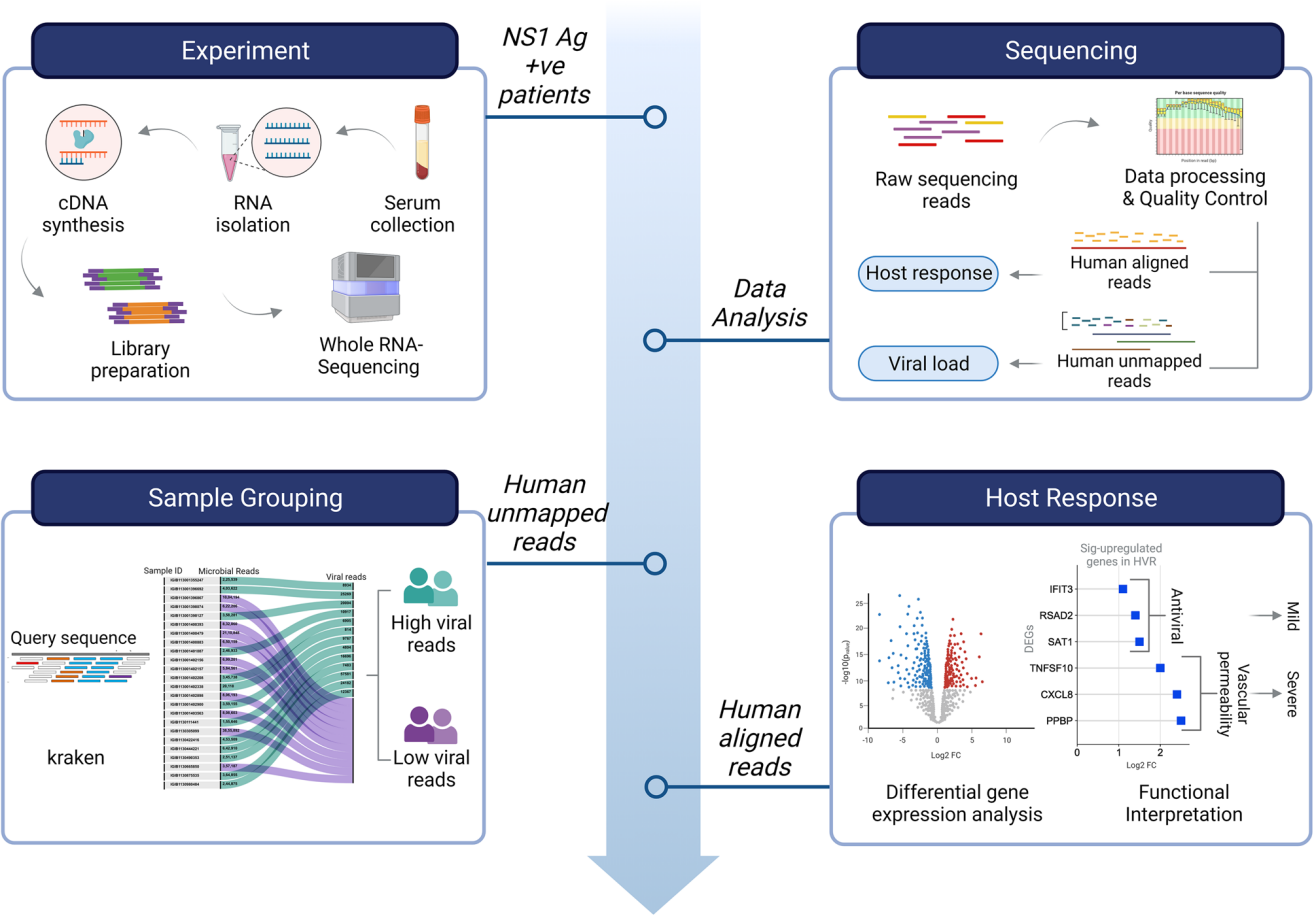
Summary of the experimental workflow and study design. The illustration highlights the key features and functional interpretation of the study wherein the RNA isolated from the serum samples of NS1 Ag positive dengue patients were used for RNA-seq followed by downstream data analysis. Using the kraken analysis, the human unmapped reads resulted in two groups: high viral reads (HVR) and low viral reads (LVR), while the human mapped reads were used for analysing the host response modulating dengue infection across the two identified groups. Figure were created with licensed version of BioRender (https://www.biorender.com/).

Our interest relied on understanding the host response from the serum during the initial stages of infection when a patient first reports to hospital with febrile illness. Serum samples collected from the patients for NS1 antigen testing, served as the source for extraction of RNA to carry out RNA-Seq for transcriptome analysis. A total of 173,169,732 raw sequencing reads were generated through sequencing of 24 Dengue positive RNA samples. Dual RNA-seq/ holo-transcriptome analysis has the advantage to deliver insights into the pathogen/microbial transcribed genome along with the human mapped reads. Henceforth, post RNA-seq, the human genome aligned reads were taken forward to investigate the host response, and the microbial reads acquired for each patient were used to study the Dengue virus features. Interestingly, although all 24 patients were NS1 antigen positive for Dengue infection, yet, the kraken2 output result for microbial reads, identified dengue virus in 12 samples, giving us the prerogative to differentiate the 24 samples as high viral reads (HVR) (n = 12) and low viral reads (LVR) (n = 12). Functional interpretation of differentially expressed genes was done to study host response. A schematic showing an overview of the study design, experimental procedure, analysis, and key findings is illustrated in Fig. 1.

### Clinical parameter distribution and trend across the dengue patients

The NS1 antigen detection in patients was accompanied with investigation of complete blood count (CBC). It is imperative to investigate the CBC in dengue as the parameters tend to change by the onset of fever, starting with leukopenia followed by thrombocytopenia and haematocrit rise if the infection progresses towards severity. We comprehensively looked at the blood profile for any early indications for patients denoting gradual shifts in laboratory parameters which might help us to understand the initial signature for confirmed Dengue infections. The blood profile included Hb (Haemoglobin), TLC (Total Leukocyte count), RBC count, MCV (Mean Corpuscular Volume), MCH (Mean Corpuscular Haemoglobin), MCHC (Mean Corpuscular Haemoglobin Concentration), Platelet count, MPV (Mean Platelet Volume), RDW (Red Cell Distribution Width), Neutrophils, Lymphocytes, Monocytes, Eosinophils, Basophils and Haematocrit. We determined normal values for all the parameters from the WHO guidelines, and then segregated the blood profile values based on whether they fell in the normal range or were lower/higher than the normal value range. We classified them as low and elevated groups. Table 2 demonstrates the percentage of patients showing low, normal or high range for the above mentioned blood parameters. Overall, the majority of the blood profile fell in the normal range group. In the low group, none of the patients showed low values for RDW, Monocytes and Basophils. Notably, Lymphocytes, Haematocrit and Eosinophils showed low values in approx. 50% of the patients. Similarly, in the normal group, only Eosinophils and lymphocytes were less than 50%, rest of the parameters were in normal range in more than 50% of patients.

**Table 2:** Distribution of laboratory blood characteristics in Dengue NS1 positive patients, with normal range for female (F), male (M) and children (C).

Parameter	Low %	Normal %	Elevated %	Normal range
Platelet count	22.7	77.3	0	150.0–410.0
Neutrophils	10	80	10	40.0–80.0
Lymphocytes	40	45	15	20.0–40.0
Haematocrit	50	50	0	40.0–50.0
MCH	30	65	5	27.0–32.0
Monocytes	0	75	25	2.0–10.0
Eosinophils	60	35	5	1.0–6.0
Haemoglobin	25	75	0	12.0–15.0 (F) 13.0–17.0 (M) 11.5–15.5 (C)
MPV	21.1	63.2	15.8	7.8–11.2
TLC	35	65	0	4.0–10.0
RBC count	30	50	20	3.8–4.8 (F) 4.5–5.5 (M) 4.0–5.2 (C)
MCV	35	65	0	83.0–101.0
RDW	0	60	40	11.5–14.5
MCHC	5	95	0	31.5–34.5
Basophils	0	100	0	0–2.0

**Figure 2:**
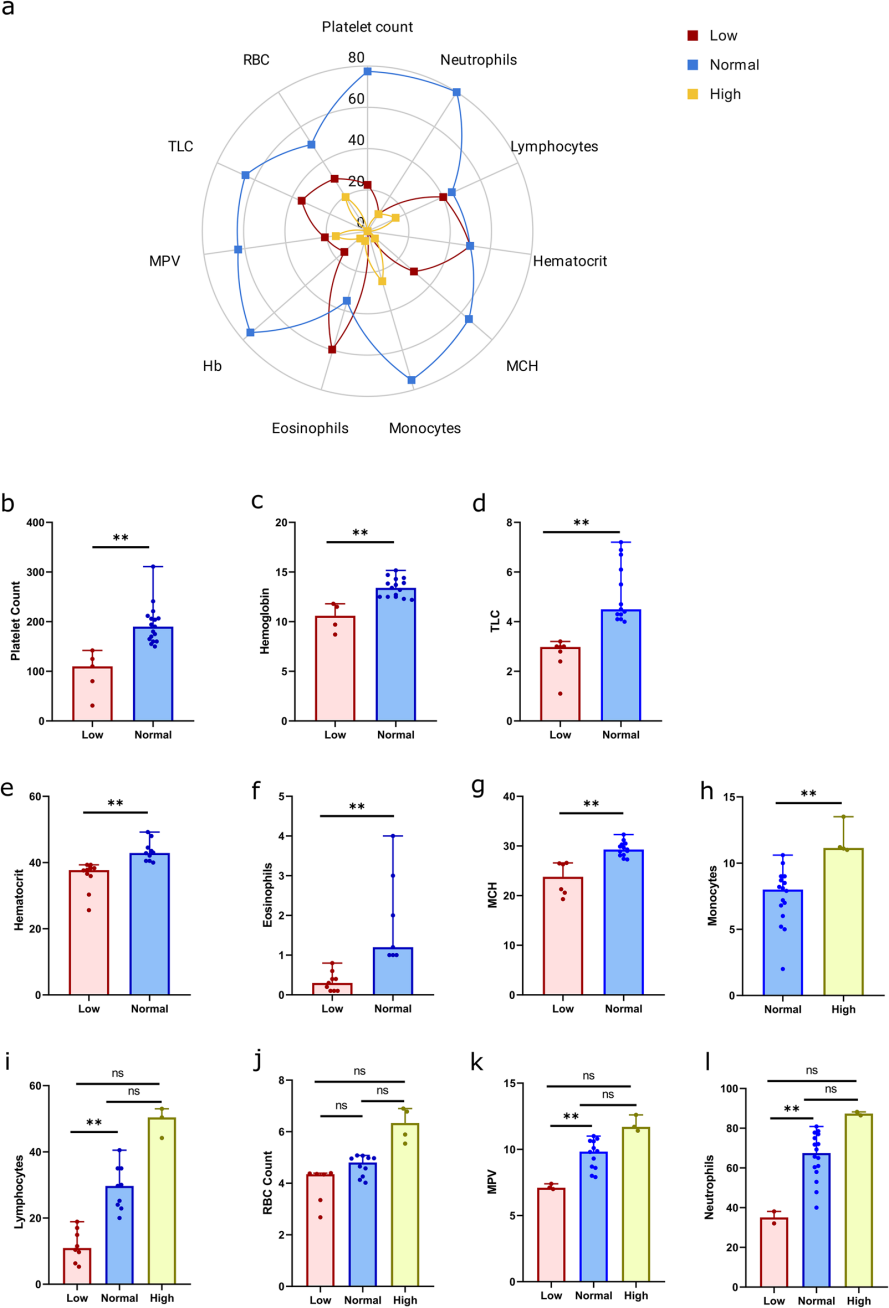
Blood parameter distribution in the study cohort. (**a**) Overall depiction of low, normal, and high values of blood profiles in the dengue infection patients. (**b**–**g**) Mann–Whitney U-test of low and normal for Platelet, Hb, TLC, Haematocrit, Eosinophils and MCH, (**h**) normal and high for monocytes, (**i**–**l**) low, normal and high for Lymphocytes, RBC Count, MPV and Neutrophils. Plots have been generated using licensed Graph pad prism version 8.

Elevated levels of blood parameters were not a common finding during the initial CBC investigation for Dengue infection, except for RDW which was found elevated in 40% of the patients. Statistical significance applied to low-normal-elevated groups using Mann–Whitney U-test, showed significance between low and normal groups for Platelet count, Hb, TLC, Haematocrit, Eosinophils, MCH, Lymphocytes, MPV and Neutrophils. Significance between normal and high groups was only detectable for monocytes. Thus, the majority of the initial clinical parameters showed near normal distribution amongst the NS1 positive Dengue patients, whereas Eosinophils, Lymphocytes (low) and RDW (high) were the ones which showed significant variability/diversion from the normal range. Intriguingly, few initial blood parameters like Neutrophils, Lymphocytes, RBC count and MPV showed values outside the normal range at both ends, low as well as high with at least 10% of patients falling in each range (Fig. 2). Hence, we furthered our investigation to look if these variabilities in initial blood profile could be addressed/inferred through integrative genomic analysis of host and pathogen through dual transcriptomics/ RNA-Seq.

### Integrative genomics approach address the variability in initial Dengue clinical parameters, viral load, and serotype

Integrative genomics approach involving host and pathogen has been known to fetch indispensable insights on host immune responses and clinical outcome in infectious diseases. We tried to address the clinical variability observed in Dengue positive patients through inferences based on integrative genomics. NS1 Ag positive based inference identified all 24 patients as Dengue virus infected.

**Figure 3:**
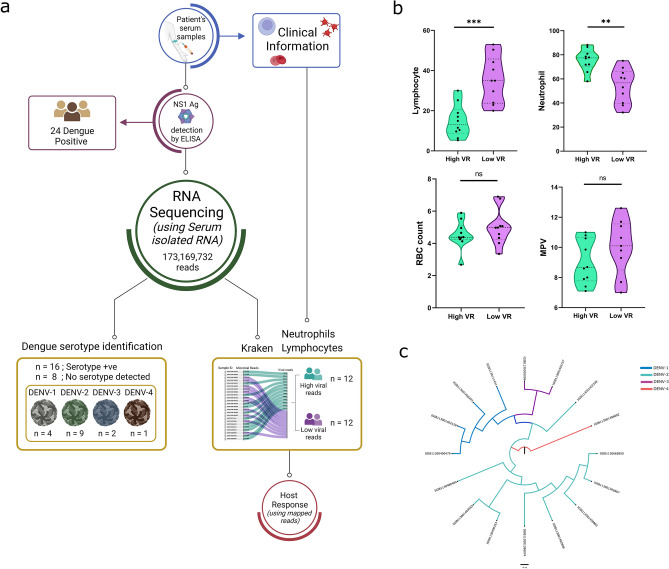
Dual RNA-seq based affirmation of the Dengue viral load and serotype. (**a**) Illustration of integrative genomics approach to understand virus and the host. Figure created with licensed BioRender (https://www.biorender.com/). (**b**) Violin plots of lymphocytes, neutrophils, RBC count and MPV, that showed both high and low variance, with respect to the two groups, showing significant difference (represented by “*”). Plots were generated using licensed Graph pad prism version 8. (**c**) Phylogenetic tree for the 16 samples for which dengue serotypes were detected. The tree was generated using locally installed MEGA 11 tool.

Dual RNA-Seq gave the transcriptomic profiles of host (human reads) as well as of microbes (non-human reads). These microbial reads were analysed through Kraken2, a *k*-mer based, taxonomic classification tool, for identification of microbes. The Kraken output file revealed a diverse group of bacteria and viruses for each of the samples (Supplementary File S1). Intriguingly, 12 out of 24 samples showed dengue viral reads domination (Sankey plot: output of kraken analysis, Supplementary File S1). The remaining 12 samples had very low dengue viral reads, hence the Sankey plot did not show presence of Dengue virus, possibly alluding to the sensitivity of the kraken based Sanky plot. The average dengue viral reads displayed by the kraken analysis tool for the samples with high dengue viral load was 712,087.3 whereas those samples with low dengue viral load had average viral reads as 515.7. Based on genetic analyses, the 24 samples could now be segregated into two groups, LVR and HVR with 12 samples each (Fig. 3a). Table 3 represents the demographic and clinical parameters of age, gender, serotype and NS1 antigen ratio for the low and high viral read groups. As evident, none of the parameters display notable difference, except for dengue serotype, where 7/12 patients of HVR were infected with serotype 2. Next, we inferred the clinical data, especially lymphocytes, neutrophils, RBC count and MPV which showed both high and low variance, with respect to the two groups. We found that Lymphocytes were significantly low and Neutrophils were significantly higher in the HVR when compared with LVR, whereas RBC count and MPV showed no significance (Fig. 3b, Table 3). Thus, an association could be seen with high Dengue viral read and low lymphocyte/high Neutrophil count in the Dengue positive patients.

**Table 3:** Demographic and clinical parameters of HVR and LVR Dengue patients.

Parameters	HVR (n = 12)	LVR (n = 12)	p value
Age, median (IQR)	25(39–65)	36(24–56)	0.088^a^
Gender, F/M	5/7	3/9	0.386^b^
Viral serotype, (DENV-1/DENV-2/DENV-3/DENV-4/unknown)	3/7/0/2/0	1/2/1/0/8	–
NS1 Antigen ratio in serum, median	3.4	3.5	0.841^a^
Lymphocytes, median	13.2	35	**0.001**^**a**^
Neutrophils, median	77.4	56.7	**0.002**^**a**^
RBC count, median	4.4	4.9	0.347^a^
MPV, median	8.65	10.1	0.522^a^

Statistical significance calculated using ^a^Mann Whitney U test and ^b^Chi^2^ test. Values of significance are highlighted in bold.

Further, Serotypes of DENV virus were identified in 16 out of 24 samples, their coverage, depth and reads information are listed in Supplementary table S1. For the remaining 8 samples, dengue serotype could not be detected. The majority (n = 9) of the genomes belonged to DENV-2 serotype, followed by DENV-1 (n = 4). Only 2 and 1 samples belonged to DENV-3 and DENV-4 serotypes respectively (Fig. 3a). As far as serotype distribution among different clinical forms is considered, all the 12 samples with high DENV reads were serotype positive, whilst only 4 samples from the LVR were positive for serotype identification. The phylogenetic distribution of the serotypes is illustrated in Fig. 3c.

### Early host response to dengue infection involved increased antiviral immune response in patients with high viral reads

**Figure 4:**
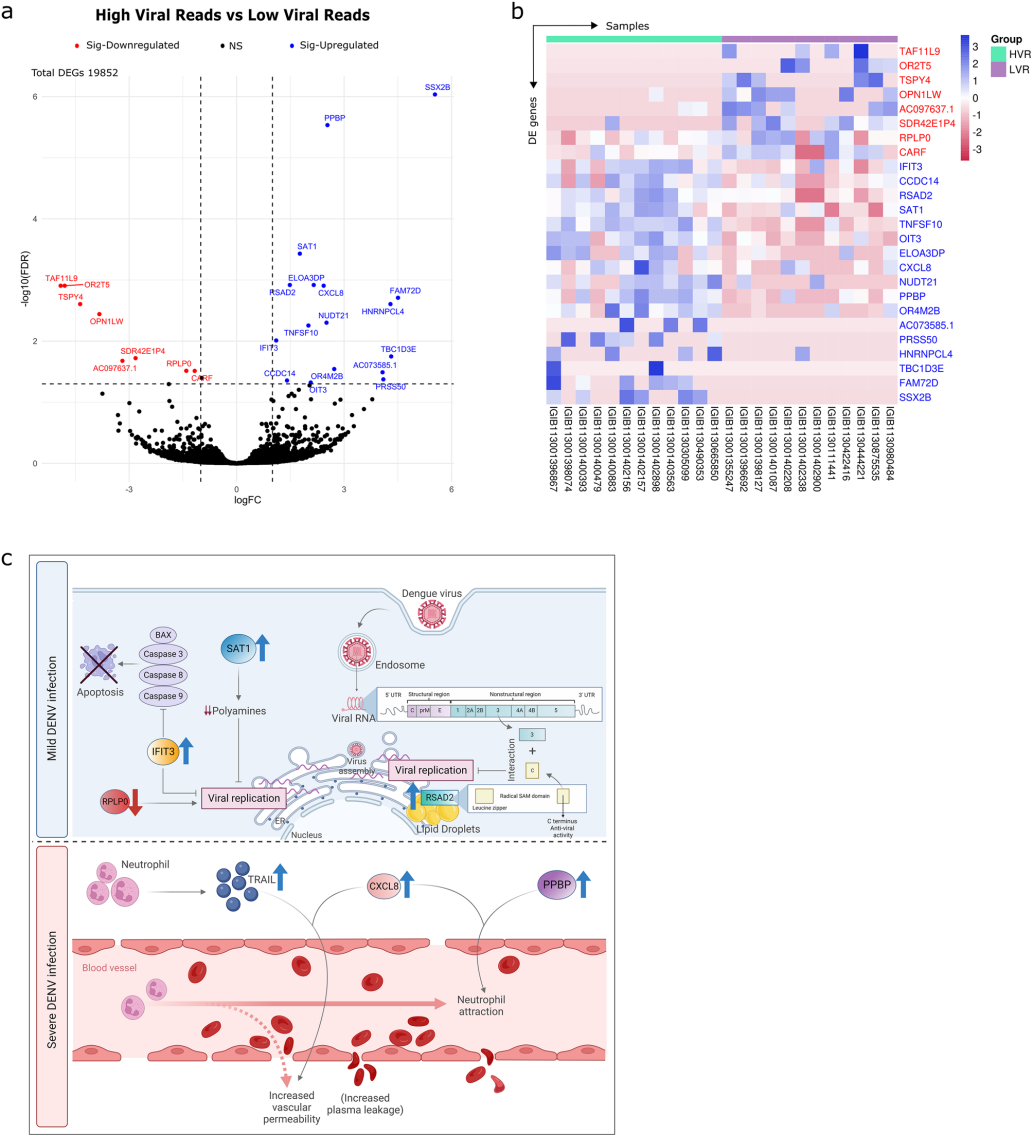
Differential gene expression across the two different viral load groups and functional interpretation of the host transcriptome profile. (**a**) Volcano plot representing differentially expressed genes highlighting the genes with log fold change of ± 1 and FDR < 0.05, generated using ggplot2 R package. (**b**) Heatmap of the 25 significant DEGs on log2 scale of normalised counts per million, created using a web tool, SR Plot (https://www.bioinformatics.com.cn/). (**c**) An overview of the possible mechanism of early host response, shifting the course of dengue infection towards mild or severe disease. Blue arrows indicate upregulated genes and red arrow indicates downregulated gene in the HVR group. Figure created with licensed version of BioRender (https://www.biorender.com/).

To identify the transcriptional signature associated with initial stages of dengue infection and characterize the immune responses to dengue virus, we compared the transcriptome data for the two dengue groups, HVR and LVR, representing differences in dengue viral reads. A total of 19,852 human differentially expressed genes (DEGs) were detected, with 25 genes being significantly differentially expressed between the groups (Supplementary table S2). The volcano plot represents the DEGs with fold change (FC) (log2FC) of >|1| and FDR value of 0.05 (Fig. 4a). We observed a significant elevated expression of 17 genes and a downregulation of eight genes in the HVR group (Fig. 4b).

Several genes related to antiviral immune response, *IFIT3, RSAD2* and *SAT1* were significantly upregulated in the HVR patients compared to patients having LVR. *RSAD2* (Viperin) is an interferon-stimulated gene, whose expression has been shown to inhibit viral infections, including influenza A virus (IAV), human cytomegalovirus (HCMV), chikungunya virus (CHIKV), and hepatitis C virus (HCV). It localizes to the cytoplasmic face of the ER as well as lipid droplets and mediates its antiviral activity against several members of the Flavivirus family, including both in vitro and in vivo mouse model of DENV infection^18^. *SAT1* (spermidine/spermine-N1-acetyltransferase), a downstream target of type I IFN stimulation, performs its antiviral role by reducing the cellular content of polyamines. Polyamines are small, positively charged molecules involved in the replication cycle of several RNA viruses, including ZIKV and Dengue.

Yet another gene, *PPBP* (pro-platelet basic protein), a potent chemoattractant and activator of neutrophils with antimicrobial properties, was found to be upregulated in the HVR groups. This might explain the increased neutrophil level in this group. Upregulation of *TNFS10* (TRAIL) grasps the attention which is known for its dual function as a rapid, apoptotic gene that facilitates host defence versus a sustained, proinflammatory function that contributes to vascular dysfunction. Elevated expression of *TNFS10* is already documented in Dengue infection with diverse functions implying the importance of this molecule towards mild disease course or severity leading to DHF^19^. Amongst the cytokines and chemokines found elevated in DENV infection, we found C-X-C motif chemokine ligand 8/interleukin-8 (*CXCL8/IL-8*) in our set of DEGs, reiterating the role of inflammatory mediators in increasing vascular permeability and dengue severity. A study has suggested that low levels of *CCL5* and high levels of *CXCL8* during early dengue infection could serve as a marker for severe dengue disease^20^. Together, the functional analysis reflects the heightened and concerted antiviral mechanisms to clear the virus as well as expression of genes that might be regulating the shift towards mild or severe disease outcomes. Figure 4c is a mechanistic illustration of the several genes with significant role during initial host response to dengue infection.

With regards to the LVR group, we found *RPLP0*, a ribosomal protein, to be significantly downregulated in that group. This gene has been shown to be required for efficient DENV-2 infection indicating decreased viral replication, thus lower reads/load observed in this group^21^. A knockdown study has revealed the role of RPLP0 for effective replication of viruses.

Additional genes were observed with significant differential expression across the two groups whose clinical relevance could not be ascertained through literature. Few olfactory receptor family genes, *OR2T5* and *OR4M2B* were present in the dengue patients, with no evidence present in the literature for dengue. *TSPY4, OPN1LW, CARF, CCDC14, HNRNPCL4, SSX2B* genes need further exploration as it has not been identified in any infectious disease, including the recent COVID-19 pandemic. Thus, it can be further elucidated for its putative role in dengue infection.

## Discussion

Dengue has an epidemic pattern and it is a cause of concern in India, due to the number of infections, differential disease severities, seasonal occurrence and revisiting crisis in health care systems every year. With lessons learnt from the COVID-19 pandemic, it is extremely important to make efforts toward pre-emptive disease understanding, especially differential disease severities, for timely healthcare and medical response preparedness. Despite numerous efforts to unravel the DENV pathogenesis, the distinguishing factors that characterize the host response to infection with the virus are largely confounding. Our primary objective was to elucidate through this study, the clinical prognostic markers and early host response observed in individuals who tested positive for NS1 Ag and hence dengue infection, which can provide leads for severe illness in patients.

An overall evaluation of the CBC of the patients with NS1 positivity, reflected the blood parameters to maximally fall in normal range with few distributions towards high and low values. Only the RDW was found to be elevated in 40% of patients. Although there are studies that reported high RBC levels in dengue patients as a hallmark of infection's progression, hardly a few studies—including one by Melissa E Day et al.—show an increase in RDW in patients with severe DF^22,23^. Contrarily, Haematocrit levels were lower or normal at this stage, which is consistent with the findings suggesting that these blood parameters begin to rise later during infection. Furthermore, TLC levels were found to be lower in 35% of dengue-infected patients, indicating the development of leukopenia, which is a common feature in Dengue patients.

Interestingly, the RNA-Seq data revealed that although all patients were serum NS1 Ag positive yet their dengue viral loads in the serum could be substantially different. Hence, we challenged ourselves to understand the initial host response signature associated with high dengue viral reads in patients compared to patients with low viral reads. It was important to note that differences in demographic parameters such as age and gender among the aforementioned groups were not statistically significant yet notably the majority of dengue patients with high viral reads were infected with DENV-2 serotype, which have been recorded with a more severe disease than other serotypes. Importantly, the initial clinical and laboratory blood parameters did provide leads when we segregated patients based on high and low viral reads. Of note, a remarkable difference in the neutrophils (high) and lymphocytes (low) counts served as important blood markers of clinical significance for the HVR. A recent review by Agata Buonacera et al. highlighted the role of neutrophil-to-lymphocyte ratio (NLR), which is calculated as a simple ratio between the neutrophil and lymphocyte counts measured in the blood, in various diseases, including sepsis, pneumonia, COVID-19, cancer, etc.^24^. Various studies have highlighted that change in levels of neutrophils and lymphocytes can serve as a robust prognostic marker of disease severity, augmenting the finding of our study in dengue infection.

Moreover, we could integrate these findings with the host gene signature and immune response, to understand the progression of the dengue infection towards severity. The transcriptomic profile of HVR compared with LVR highlighted two aspects governed by different sets of genes. Firstly, heightened expression of antiviral genes which might elicit an interferon/immune response to fight the spread of the dengue virus in the host. The IFN-I response represents the principal effector mechanism of innate immunity for the control of DENV replication^25^. The presence of antiviral genes reiterated that the host is actively elevating its defence mechanisms in response to high dengue viral load. Interestingly, *RSAD2* (Viperin), identified as an upregulated gene in the HVR group, has been demonstrated to interact with the NS3 protein of dengue virus mediating anti-dengue activity via its C-terminus^26^. It is important to emphasise that although Viperin has been earlier demonstrated to have an antiviral role, this is the first study to highlight its role of modulating dengue host response using clinical samples. *IFIT3* was another highly enriched gene having an antiviral effect on dengue virus^27^. Deficiency of *IFTI3* is associated with increased viral production, along with increased rates of apoptotic cell death as it has been shown to inhibit the apoptotic regulators such as caspase 3, caspase 8, caspase 9, and Bcl-2-associated X protein (BAX)^28^. As our lab has investigated the host response to SARS-CoV-2 infection, it seems reasonable to mention here that, in COVID-19 disease, interferon response genes were rarely captured in our study, in contrast to Dengue, where the major DE genes between HVR and LVR groups were those of interferon response.

Parallelly, the activation of immune responses can lead to increased release of cytokines, a phenomenon also observed in our study. This phenomenon has the propensity to increase the risk of vascular permeability in dengue infections and has been proposed to play an important role in the pathogenesis of severe dengue^29^. Upregulation of genes such as *CXCL8* (platelet cytokine) and *TNFSF10* (apoptosis mediator) in HVR at an early stage of infection might act as pointers of a severe disease course. These soluble factors have been reported to be higher in patients with severe dengue compared to those with mild form of the disease^30^. Recently, *TNFSF10* (TRAIL) is reported as an emerging novel angiogenic factor that shares functional overlap with *VEGF* for vascular permeability, leukocyte extravasation and angiogenesis^19^. *VEGF* has shown a pivotal role in mediating plasma leakage in dengue as evidenced by its elevated levels associated with DHF and/or DSS^31,32^). The increased expression of *TNFSF10* and *CXCL8* in our patient cohort can be correlated clinically to lymphocyte and neutrophil counts in dengue HVR patients. *CXCL8* is considered the most potent neutrophil chemoattractant during inflammation^33^ whereas neutrophils are also reported to secrete *TNFSF10* as defence against bacterial pathogens, as documented during Asthma^34^. Moreover, *TNFSF10* is also associated with functional maturation of lymphocytes and homeostasis^35^. Hence, Lymphocyte count reduction in patients suffering from high viral reads can be a prognostic factor for downstream severity since lymphocytes actively participate in warding off pathogens essentially through the adaptive immune system^36–38^. Furthermore, the high viral read group had an increase in neutrophils, indicating the activation of inflammatory responses in the host, as mentioned above.

Learning from the differential disease trajectory/symptoms during and after COVID-19 globally as well as multitude of studies from our Lab, wherein we intended to understand the human host response to SARS-CoV-2 using the RNA isolated from nasopharyngeal swab samples, we used the same sample source for SARS-CoV-2 detection as well as RNA-Seq to study the host transcriptome^39,40^. Herein, in order to identify the early predictor of dengue disease severity, we employed the RNA isolated from dengue patients’ serum samples, which is used for dengue NS1 Ag testing. A similar study approach can be applied for other RNA viruses, such as the arboviruses Chikungunya virus (CHIKV) and Zika virus (ZIKV), which have both re-emerged globally. For these viral diseases, the RNA-seq based approach can be used to identify the early host signatures from the serum/plasma and blood respectively, which are the source for diagnosing virus infection.

## Conclusion

**Figure 5:**
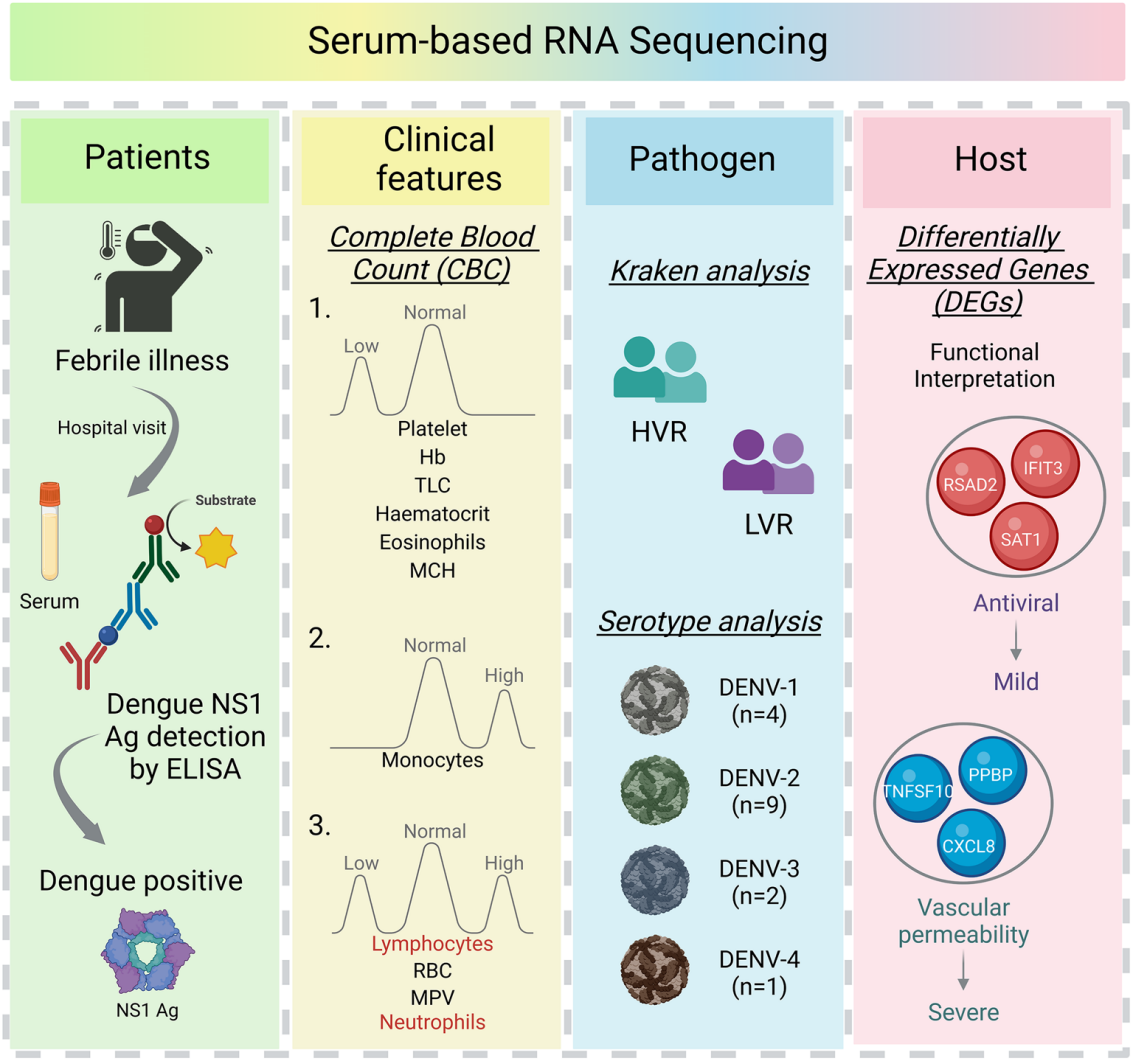
Elucidates the integrative approach wherein serum samples are used for dual RNA-Sequencing of the dengue NS1 Ag positive patients, with CBC profile highlighting Lymphocytes (low) and Neutrophils (high) as important clinical parameters, and the early host response to dengue virus. Figure created with licensed BioRender (https://www.biorender.com/).

This is the first study which presents the serum RNA based early host transcriptomic response to dengue infection, when patients first report to clinics for Dengue NS1 Ag testing. We highlighted low lymphocytes and high neutrophils as initial blood markers which were associated with high dengue viral reads in NS1 positive patients. Interestingly, although all patients were NS1 positive, yet dengue viral loads were not observed uniformly amongst the patients. Transcriptomic analysis revealed upregulation of important antiviral genes which could be conferred as an attempt of the host to fight disease in the high viral read group of patients. Yet, increased expression of few soluble mediators at an early stage of infection reiterates their plausible function in digressing the disease course towards severity in due course of time (Fig. 5). Together these findings can provide leads for identifying early markers during CBC profiling as well as serum RNA samples, which could predict disease course and hence help patients’ segregated handling during surges in Dengue infection in India.

Lastly, we also want to highlight that this study was carried out as a pilot study, to understand the pathogen load and the host response to it from the same serum sample, which was used for NS1 testing in a set of 24 patients. This small sample size might be considered a limitation to the present study, but we intend to widen our research in a subsequent study involving a large cohort of dengue patients, with clinical parameter based severities. Another limitation to the study can be lack of longitudinal sampling, which would have strengthened our findings, but acknowledging that these are clinical samples of patients showing at OPD of a hospital, we appreciate the importance of time sensitive understanding of the disease dynamics.

## Materials and methods

### Study design, sample collection and RNA isolation

The study was carried out at CSIR-Institute of Genomics and Integrative Biology (CSIR-IGIB) in collaboration with MAX Healthcare Hospital, Delhi, India. Serum samples from dengue positive patients were obtained from MAX Hospital. The serum collection and Dengue NS1 Antigen Test (ELISA) to detect dengue was done by the paramedical staff at the hospital on the day of reporting, by trained medical staff with required safety precautions using Bio-Rad Platelia Dengue NS1 Ag test. Viral RNA was isolated from the collected serum using QIAamp RNA Blood Mini Kit (cat. No. 52304). The study design segregated the 24 dengue NS1-positive patients into two groups of 12 patients each as having Low and High Dengue viral reads, based on kraken2 output.

### Library preparation and sequencing

A total of 250 ng of total RNA were taken for library preparation using Illumina TruSeq Stranded Total RNA Library Prep Gold (Illumina, Cat. No. 20020598) as per manufacturer’s reference guide (1000000040499 v00). The ribosomal RNA (both cytoplasmic and mitochondrial) were first removed using biotinylated target specific oligos followed by fragmentation. First strand cDNA synthesis was carried out from the cleaved RNA fragments using reverse transcriptase and random primers, followed by second strand synthesis using DNA polymerase 1 and RNase H. This double stranded cDNA has been purified using AMPure XP (Beckman Coulter, A63881) followed by adenylation of 3ʹ blunt end of the double stranded cDNA. Each library was tagged with unique indexes which were then enriched by PCR amplification. Subsequently, the final library was purified using AMPure XP (Beckman Coulter, A63881) and quality of libraries checked using Agilent 2100 Bioanalyzer. Final loading concentration of 650 pM was used for sequencing, performed on the NextSeq 2000, with paired end 2 × 151 read length.

### Clinical segregation and statistical analysis

All metadata of 24 Dengue positive patients, including gender, age, and Complete blood profile/Count (platelet count, neutrophils, lymphocytes, hematocrit, MCH, monocytes, eosinophils, haemoglobin, MPV, TLC, RBC, MCV, RDW, MCHC, and Basophils) were collected from electronic medical records and used for statistical analysis. Statistical analysis for clinical parameters were done through Mann–Whitney test using Graph pad prism version 8.

### RNA pre-processing and host transcriptome analysis

Raw sequencing reads were converted to FASTQ format using bcltofastq. For each sample, quality checks were done using FASTQC, and adapter trimming using Trimmomatic-0.36 to remove low quality reads^41^. The high quality reads contained both human and microbial reads. Next, the trimmed FASTQ were mapped/aligned to the reference human transcript^42^ using salmon 0.21^43^. Reads were aligned to reference human transcript as a pseudo-alignment by salmon. Salmon provides fast and accurate transcript expression quantification. Further while annotating the transcripts to genes to know Gene Expression, we have used reference human genome. The reference human genome taken was GrCh38 (Gencode v40). Differential genes were identified based on counts obtained from sequence alignment files generated by salmon, which were imported into the R environment using tximport^44^. Count based differential gene expression analysis was performed using EdgeR tool^45^. Counts were normalised and scaled to account for library size using the TMM (trimmed mean of M values) approach. Only counts that fell greater than equal to 2 gene counts per million reads (CPM) were kept. For the selection of DE genes, a false discovery rate (FDR) adjusted p-value < 0.05 was used with fold change of greater than or equal to ± 1. ggplot2 R package was used to visualize the data as a volcano plot. For the heatmap, significant differential expression genes were taken and visualised using a web tool, SR Plot^46^.

### Meta-transcriptomic analysis

In addition, for the microbial reads, Kraken2^47^ was performed to identify the Dengue reads across the samples, using the non-human reads from the dual RNA-seq data^48,49^. Briefly, Kraken2 is a taxonomic classifier for mapping of taxa using k-mers from the genomic database and assigning microbial communities to the reads. The kraken2 database contains bacteria, archaea and viral reference sequences.

### Mapping to pathogen genome and serotype classification

Trimmed Fastqs were mapped to DENV Serotypes 1–4, using HISAT2^50^. The final serotype of a particular sample is determined by the serotypes with the highest percentages in that sample. BAM was converted into consensus FASTA using bcftools^51^. Further, to know the diversity among these serotypes, phylogenetic analysis was done by concatenating the fasta sequences of all the samples and uploaded in MEGA 11^52^ and phylogeny was done using maximum-likelihood method.

### Ethics approval and consent to participate

The study was approved by the Institutional Ethics Committee of both CSIR-Institute of Genomics and Integrative Biology, and Max Super Speciality Hospital, under the approval number CSIR-IGIB/IHEC/2020-21/01. Written consent was taken from the participants for enrolment in the study. All experimental methods and clinical sampling were performed in accordance with the relevant guidelines and regulations.

### Supplementary Information

Supplementary Information 1.

Supplementary Table 1.

Supplementary Table 2.

## Data Availability

The datasets generated during the current study are available in the NCBI SRA under the access number PRJNA955953.
