# Supplementary material for: Early transcriptomic host response signatures in the serum of dengue patients provides insights into clinical pathogenesis and disease severity
Source: Sci Rep. 2023 Aug 29;13:14170. doi: 10.1038/s41598-023-41205-2 (PMC10465479; doi:10.1038/s41598-023-41205-2)
Supplement: Supplementary file 1 — Supplementary Information 1. [file 41598_2023_41205_MOESM1_ESM.docx]

**Supplementary File 1:** Kraken output file showing a diverse group of bacteria and viruses for all 24 samples, with 12 samples having high dengue virus reads.
